# Supplementary material for: p62 sorts Lupus La and selected microRNAs into breast cancer-derived exosomes
Source: bioRxiv. 2025 Mar 20:2025.03.20.644464. Preprint. [Version 1] doi: 10.1101/2025.03.20.644464 (PMC11957149; doi:10.1101/2025.03.20.644464)
Supplement: 1 [file NIHPP2025.03.20.644464V1-supplement-1.pdf]

bioRxiv preprint doi: https://doi.org/10.1101/2025.03.20.644464; this version posted March 20, 2025. The copyright holder for this preprint (which was not certified by peer review) is the author/funder, who has granted bioRxiv a license to display the preprint in perpetuity. It is made available under aCC-BY 4.0 International license.

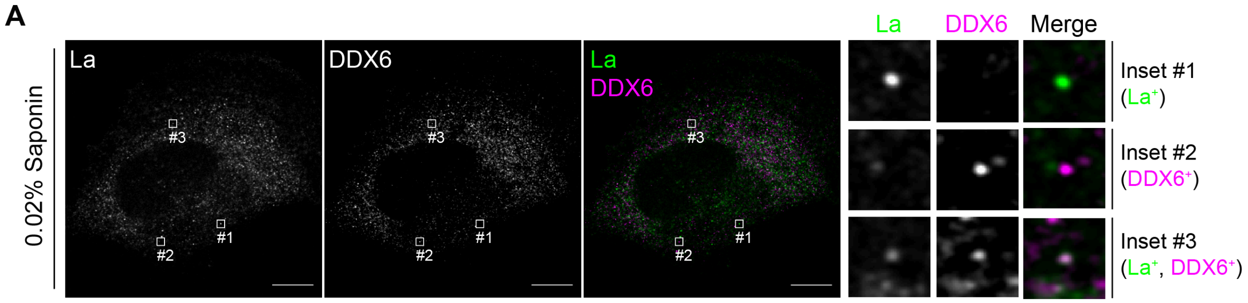

Figure S1. Co-localization of La with the P-body marker, DDX6.

A. Airyscan microscopy of endogenous La and DDX6 from MDA-MB-231 WT cells permeabilized with 0.02% saponin. Insets indicate La-positive, DDX6-positive and La/DDX6 double-positive puncta. Green: La; Magenta: DDX6. Scale bar: 10 μm.

bioRxiv preprint doi: https://doi.org/10.1101/2025.03.20.644464; this version posted March 20, 2025. The copyright holder for this preprint (which was not certified by peer review) is the author/funder, who has granted bioRxiv a license to display the preprint in perpetuity. It is made available under aCC-BY 4.0 International license.

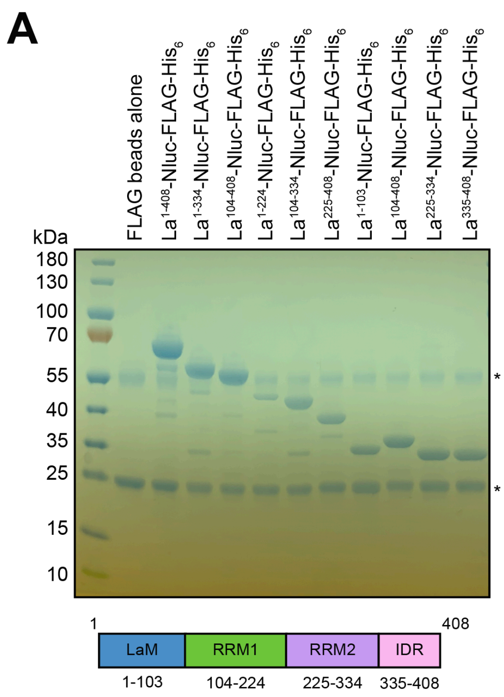

Figure S2. Purification of recombinant La proteins.

A. Coomassie-stained SDS-PAGE gel showing anti-FLAG bound full-length and truncated La proteins prior to elution using 3xFLAG peptide. Asterisks (*) indicate anti-FLAG antibody fragments that were eluted from the resin upon denaturing elution with 1X Laemmli buffer (reducing).

bioRxiv preprint doi: https://doi.org/10.1101/2025.03.20.644464; this version posted March 20, 2025. The copyright holder for this preprint (which was not certified by peer review) is the author/funder, who has granted bioRxiv a license to display the preprint in perpetuity. It is made available under aCC-BY 4.0 International license.

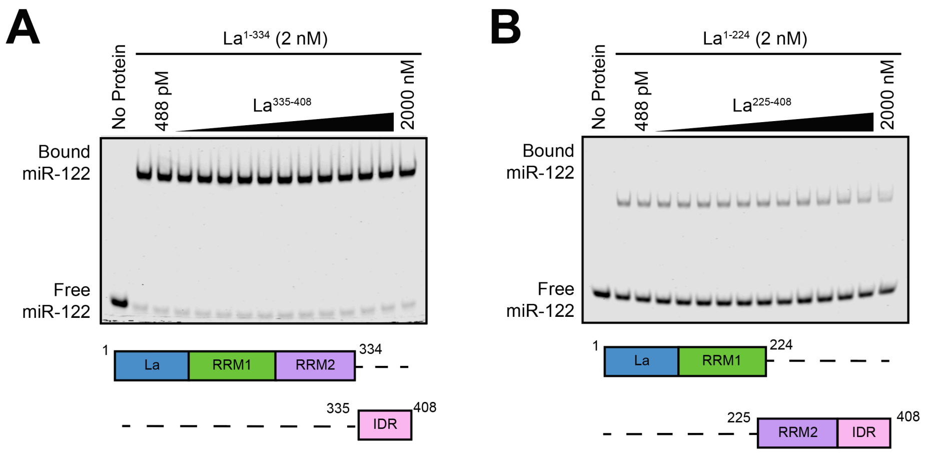

Figure S3. Competition EMSAs between truncated La proteins.

A. EMSA with 5' fluorescently labeled miR-122, 2 nM La$^{1-334}$ and increasing concentrations of La$^{335-408}$. La$^{335-408}$ was titrated from 448 pM to 2 µM. miR-122 migration was detected using in-gel fluorescence. **B. .** EMSA with 5' fluorescently labeled miR-122, 2 nM La$^{1-224}$ and increasing concentrations of La$^{225-408}$. La$^{225-408}$ was titrated from 448 pM to 2 µM. miR-122 migration was detected using in-gel fluorescence.

bioRxiv preprint doi: https://doi.org/10.1101/2025.03.20.644464; this version posted March 20, 2025. The copyright holder for this preprint (which was not certified by peer review) is the author/funder, who has granted bioRxiv a license to display the preprint in perpetuity. It is made available under aCC-BY 4.0 International license.

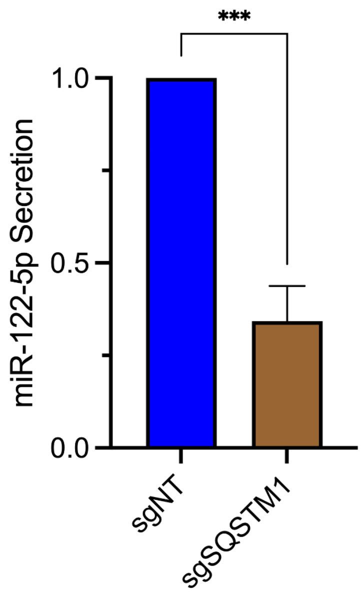

Figure S4. p62 is required for the secretion of miR-122.
A. RT-qPCR analysis of miR-122 from high-speed pellet fractions isolated from the conditioned medium of MDA-MB-231 cells expressing HA-CD63-mEGFP^{ECL1}, ZIM3-KRAB-dCas9 and either sgNT or sgSQSTM1.
